# Long-term outcomes of pelvic exenterations for gynecological malignancies: a single-center retrospective cohort study

**DOI:** 10.1186/s12885-024-11836-3

**Published:** 2024-01-17

**Authors:** Jie-Hai Yu, Chong-Jie Tong, Qi-Dan Huang, Yun-Lin Ye, Gong Chen, Hao Li, Yong-Shan Wen, Fan Yang, Nan-Bin Luo, Guang-Yu Xu, Ying Xiong

**Affiliations:** 1 State Key Laboratory of Oncology in South China, Collaborative Innovation Center for Cancer Medicine, Sun Yat-Sen University Cancer Center, Guangzhou, China; 2 Department of Colorectal Surgery, Sun Yat-Sen University Cancer Center, Guangzhou, China; 3 Department of Gynecologic Oncology, Sun Yat-Sen University Cancer Center, No. 651, Dongfeng East Road, Guangzhou, 510060 P. R. China; 4 Department of Urology, Sun Yat-Sen University Cancer Center, Guangzhou, China; 5 Department of Head and Neck, Sun Yat-Sen University Cancer Center, Guangzhou, China; 6 Suzhou Medical College of Soochow University, Suzhou, China

**Keywords:** Pelvic exenteration, Gynecological cancer, Survival, Surgical complication, Single-port laparoscopic surgery

## Abstract

**Background:**

Recently, with the advancement of medical technology, the postoperative morbidity of pelvic exenteration (PE) has gradually decreased, and it has become a curative treatment option for some patients with recurrent gynecological malignancies. However, more evidence is still needed to support its efficacy. This study aimed to explore the safety and long-term survival outcome of PE and the feasibility of umbilical single-port laparoscopic PE for gynecologic malignancies in a single medical center in China.

**Patients and methods:**

PE for gynecological cancers except for ovarian cancer conducted by a single surgical team in Sun Yat-sen University Cancer Center between July 2014 and December 2019 were included and the data were retrospectively analyzed.

**Results:**

Forty-one cases were included and median age at diagnosis was 53 years. Cervical cancer accounted for 87.8% of all cases, and most of them received prior treatment (95.1%). Sixteen procedures were performed in 2016 and before, and 25 after 2016. Three anterior PE were performed by umbilical single-site laparoscopy. The median operation time was 460 min, and the median estimated blood loss was 600 ml. There was no perioperative death. The years of the operations was significantly associated with the length of the operation time (*P* = 0.0018). The overall morbidity was 52.4%, while the severe complications rate was 19.0%. The most common complication was pelvic and abdominal infection. The years of surgery was also significantly associated with the occurrence of severe complication (*P* = 0.040). The median follow-up time was 55.8 months. The median disease-free survival (DFS) was 17.9 months, and the median overall survival (OS) was 25.3 months. The 5-year DFS was 28.5%, and the 5-year OS was 30.8%.

**Conclusion:**

PE is safe for patient who is selected by a multi-disciplinary treatment, and can be a curative treatment for some patients. PE demands a high level of experience from the surgical team. Umbilical single-port laparoscopy was a technically feasible approach for APE, meriting further investigation.

**Supplementary Information:**

The online version contains supplementary material available at 10.1186/s12885-024-11836-3.

## Background

Pelvic exenteration (PE) is a radical surgical procedure involving the resection of multiple endopelvic and extra-pelvic organs, originally introduced by Alexander Brunschwig in 1948 as a palliative treatment for cervical cancer patients with residual or recurrent disease post-radiotherapy [1, 2]. PE used to have a high perioperative mortality rate but failed to achieve satisfactory survival [3, 4].

In the past decades, with the optimization of candidate selecting and the advancement of medical technology, the mortality of PE has descended to less than 5%, and the 5-year survival rate could reach 20%-72.6% [5–8]. This remarkable progress has repositioned PE from a palliative intervention to a radical treatment approach for certain cases of recurrent or locally advanced gynecological malignancies, particularly in scenarios where no other equally effective treatment alternative is available. The rationale behind this transition lies in the more rigorous patient selection process, which meticulously assesses the extent of disease involvement, patients' overall health status, and potential postoperative recovery. Advancements in surgical techniques, particularly the introduction of minimally invasive approaches, have further revolutionized PE. Traditional laparoscopic and robot-assisted laparoscopic surgery have become viable surgical options for PE. These approaches aim to reduce morbidity, enhance postoperative quality of life, and improve overall outcomes [9–12].

Despite significant strides in enhancing the surgical outcomes of PE, it remains a complex and demanding procedure necessitating a high degree of technical skill and extensive experience from the surgical team. Consequently, its application has been more limited in developing countries.

Here, we retrospectively investigated the safety and long-term survival outcome of PE as well as the feasibility of minimal invasive PE for gynecologic malignancies in a single medical center in China, aiming to provide more evidence for the clinical application of PE.

## Patients and methods

### Study design

This was a single-center retrospective study. Data of patients who underwent pelvic exenteration performed by Dr. Ying Xiong between July 2014 and December 2019 at Sun Yat-sen University Cancer Center was retrospective collected and this study was approved by the Ethics Committee of Sun Yat-sen University Cancer Center (approval number: B2021-381).

### Patient selection

The main inclusion criteria for PE were: (1) Patients with pathologically confirmed gynecological cancer; (2) Tumors that recurred or remained uncontrolled after other treatments, and are confined within the pelvic cavity, or locally advanced gynecological malignancies; (3) No equally effective treatment option is available which is rigorously evaluated by multidisciplinary treatment (MDT); (4) Patient can tolerate the surgery; (5) Thorough communication with the patients and informed consent signed. Exclusion criteria included extra-pelvic metastasis assessed by physical examination or radiology, and ovary and fallopian tubal malignancies.

Patients who underwent PE with complete clinical data were included in this study.

### Surgical procedure

The procedures of resection were performed by the gynecological surgical team. After the exenteration, surgeons of urology, gastrointestinal and head and neck completed the reconstruction.

For single-port laparoscopic surgeries, after the establishment of the single-port laparoscopic operating channel, an assistant operating incision will be made in the area where ileal catheterization is expected. After the procedures of exenterations, the urologist will perform the ileal catheterization, and finally the stoma was located in the assistant operation incision.

### Data collection

Data of baseline demographics, operative details, pathology information, complication details and outcomes were collected. Complication was classified by Clavien-Dindo criteria [13]. Grade III and above were defined as severe complication. Postoperative morbidity was categorized as early (< 30 days after surgery) or late (≥ 30 days after surgery). Tumor persisted or relapsed within 6 months was considered as uncontrol, while relapsed after 6 months was considered as recurrence.

### Statistical analysis

Continuous variables were represented by the median (range), and categorical variables were described by frequency or percentage. Mann–Whitney U test, Fisher exact test, and chi-square test were used for comparison between groups as appropriate. Survival analysis was conducted using the Kaplan–Meier method. The starting time point in the current study was the day underwent the operation. Disease free survival (DFS) was defined as the absence of any recurrence, including the local site and distant recurrence, and death from any case. Overall survival (OS) was defined as the absence of death from any case. All statistical analyses were based on two-tailed hypotheses, and a *P* < 0.05 was considered statistical significant. Analyses were performed with SPSS version 22.0 (IBM, Inc., Armonk, New York).

## Results

### Baseline characteristics of participants

A total of 41 patients were included, with a median age of 53 (range 26–67) years (Table 1). Cervical cancer was the most common cancer type (87.8%), followed by vaginal stump cancer (4.9%). Only two (4.9%) patients had primary cervical cancer, while the remaining 39 cases involved uncontrolled (56.1%) or recurrent (39.0%) malignancies. Notably, most of them received prior treatment (Supplementary Table S1). As for the symptoms before PE, 9 patients (22.0%) had infection, 8 patients (19.5%) suffered from pain, and 5 (12.2%) experiencing intestinal or urinary fistulas.

**Table 1:** Baseline characteristics

Characteristics	n (%)
**Age (y)**	
Median (range)	52.5 (26–67)
**Cancer type**	
Cervical cancer	36 (87.6)
Vaginal stump cancer	2 (4.9)
Endometrial cancer	1 (2.4)
Vaginal cancer	1 (2.4)
Endometrial stromal sarcoma	1 (2.4)
**Previous treatment**	
Yes	39 (95.1)
No	2 (4.9)
**Symptoms before surgery**	
Infection	9 (22.0)
Pain	9 (22.0)
Fistula	5 (12.2)

### Operation details

**Fig. 1:**
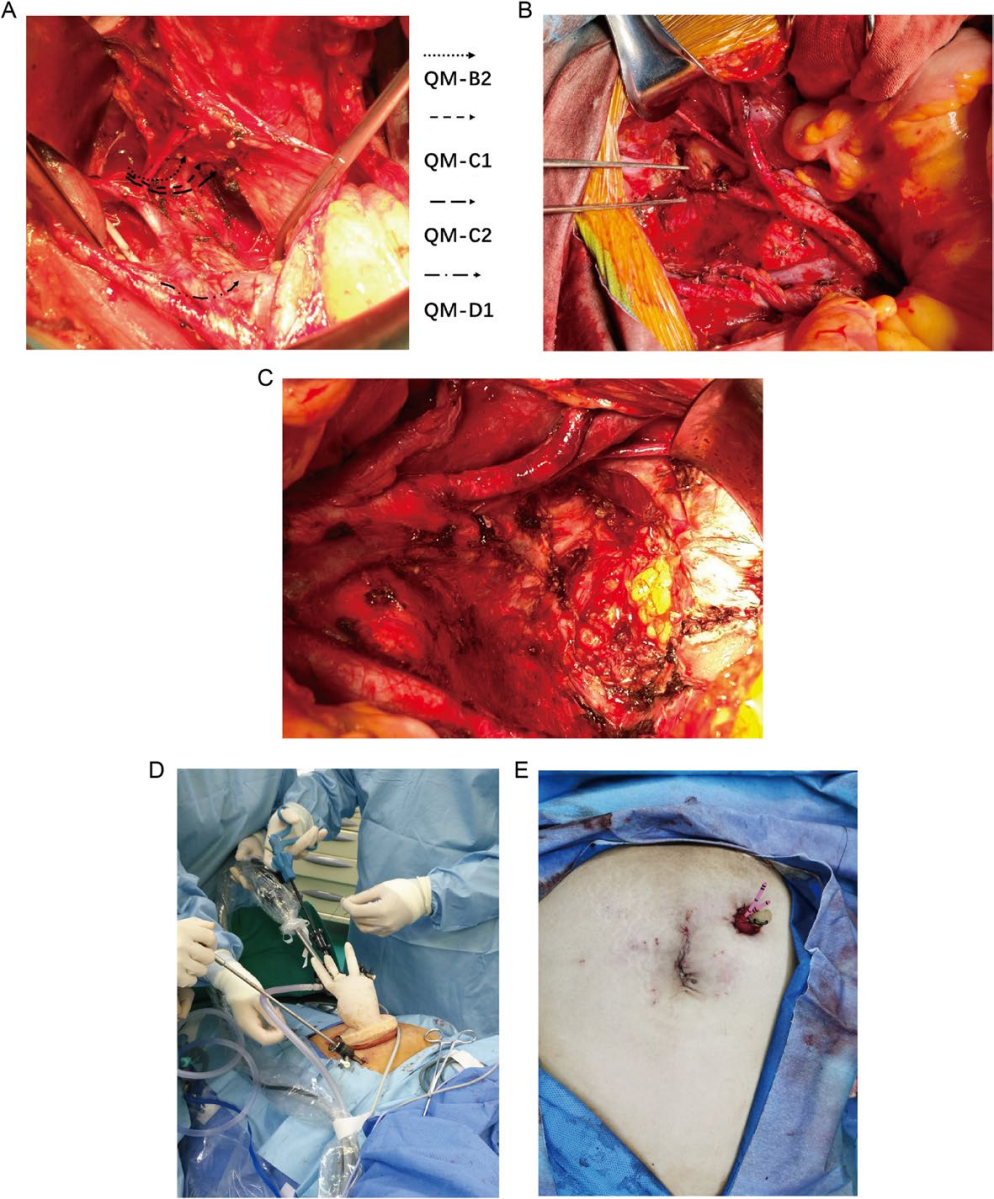
Representative surgical images of PE. **A** Indication of the resection border according to Q-M classification. **B** The resection of the pelvic side wall, and the pelvic devascularization. **C** The resection of levator ani muscle. **D** The distribution of incisions in single-port laparoscopic surgeries. **E** Surgical wound after single-port laparoscopic surgery

Forty PE were performed with a curative intention, while one case was palliative PE. Sixteen (39.0%) of the procedures were performed in or before 2016, whereas the others were performed after 2016. During the operation, abdominal and pelvic metastasis were found in 2 cases, of which 1 case had macroscopic residual tumor after the procedure. Total, anterior and posterior exenteration (TPE, APE, PPE) was performed in 30 (73.2%), 8 (19.5%), and 3 (7.3%) patients, respectively. Thirty-five supralevator PE and 6 infralevartor PE were performed respectively (Fig. 1). Eleven patients (26.8%) underwent pelvic floor reconstruction with omentum J flap formation. As for the surgical approach, three patients (7.3%) underwent trans-umbilical laparoendoscopic single site anterior exenteration (LESS-APE, Fig. 1), and the remaining 38 patients (92.7%) underwent laparotomy surgeries.

The median operation time was 460 (range 257–925) min, and the estimated blood loss was 600 (range 50–3000) ml and 34 patients (81.0%) underwent intraoperative blood transfusion. Operation time of surgeries performed in or before 2016 was significantly longer than that of surgeries conducted after 2016 (550 min vs 410 min, *P* = 0.0018). The median postoperative exhaust time was 5 (range 3–13) days, and the median postoperative hospital stay was 16 (9–69) days (Table 2). No perioperative death (within 30 days after surgery) happened in this cohort. For APE, the volume of intraoperative blood loss of patients who underwent LESS-APE was remarkably less than those who underwent laparotomy APE (183.3 ml vs 760.0 ml, *P* = 0.024). However, there was no significant difference in operation time, postoperative exhaust time and the length of the postoperative hospital stay between these two groups.

**Table 2:** Operation characteristics

Characteristics	n (%)
**Operation time (min)**	
Median (range)	460 (257–925)
**Blood loss (ml)**	
Median (range)	600 (50–3000)
**Blood transfusion (ml)**	
Median (range)	600 (50–3000)
**Hospital stays after surgery (d)**	
Median (range)	16 (9–69)
**Time of exsufflation (d)**	
Median (range)	5 (3–13)
**Operation type**	
Total PE	30 (73.2)
Anterior PE	8 (19.5)
Posterior PE	3 (7.3)
**Musculus levator ani resection**	
Yes	6 (14.6)
No	35 (85.4)
**Pelvic floor reconstruction**	
Yes	11 (26.8)
No	30 (73.2)
**Operation approach**	
Laparotomy	38 (92.7)
Single-port laparoscopy	3 (7.3)

### Pathological details

Twenty-seven patients (65.9%) underwent pelvic or (and) para-aortic lymphadenectomy, and 5 patients (18.5%) had pathologically confirmed lymph node metastasis. Seventeen cases (41.5%) had tumors larger than 4 cm. Ten patients (24.4%) had positive surgical margins of the pelvic wall, which was defined as pelvic wall involvement. Totally, 30 PE in the current study were considered as a radical one (no macroscopic residual tumor and no pelvic wall involvement), whereas the other 11 PE were defined as a palliative PE.

### Postoperation morbidities

The overall complication rate was 52.4%, and the early and late morbidities were 51.2% and 26.8%, respectively (Table 3 and Supplementary Table S2). Six severe early complications occurred in 4 patients (9.6%), and 6 major late complications occurred in 6 patients (14.6%). Patients with grade I and grade II complications were all cured. A 47-year-old patient (2.4%) died 38 days after the procedure due to septicemia and multiple organ dysfunction syndrome. A total of 8 cases (19.5%) underwent secondary surgeries due to severe complications. Three complications occurred in patients undergoing LESS-APE, whereas in patients who underwent open APE, two complications occurred.

**Table 3:** Details of postoperative complications

Complications	Early (33 cases)	Late (17 cases)	Total (50 cases)
**Morbidity**	51.2% (21/41)	26.8% (11/41)	53.7% (22/41)
**Abdominal and pelvic infections**	13 (39.4%)	5 (29.4%)	18 (36.0%)
**septicemia**	1 (3.0%)	0	1 (2.0%)
**MODS**^a^	1 (3.0%)	0	1 (2.0%)
**Intestinal obstruction**	6 (18.2%)	0	6 (12.0%)
**Hemorrhage**	1 (3.0%)	0	1 (2.0%)
**Anastomotic fistula**	2 (6.1%)	0	2 (4.0%)
**Vesicovaginal fistula**	0	1 (5.9%)	1 (2.0%)
**Ureteral fistula**	2 (6.1%)	1 (5.9%)	3 (6.0%)
**Intestinal fistula**	1 (3.0%)	3 (17.6%)	4 (8.0%)
**Rectovaginal fistula**	0	1 (5.9%)	1 (2.0%)
**Wound problems**	4 (12.1%)	2 (11.8%)	6 (12.0%)
**Thrombosis**	0	2 (11.8%)	2 (4.0%)
**Lymphocystis**	1 (3.0%)	1 (5.9%)	2 (4.0%)
**Chylous leakage**	1 (3.0%)	0	1 (2.0%)
**Perineal hernia**	0	1 (5.9%)	1 (2.0%)
**Secondary operation**	3 (7.3%)	5 (12.2%)	8 (19.5%)

^a^*MODS* Multiple organ dysfunction syndrome

We investigated potential factors influencing postoperative complications but did not identify any factors with statistically significant differences in univariate analysis (data not show). Given the comprehensive nature of PE and the high incidence rate of overall complications, we delved deeper into the determinants of severe complications. We found that the year of surgery was the only factor significantly correlated with the occurrence of severe postoperative complications (Table 4). Patients who underwent surgery in 2016 or earlier had a significantly higher incidence of severe complications compared to those who had surgery after 2016 (37.5% vs 8.0%, *P* = 0.040).

**Table 4:** Univariate analysis for severe complications after PE

Characteristic	Percentage	*P*-value
**Age, y**		
< 60	21.6%	0.569
≥ 60	0	
**History of radiotherapy**		
Yes	21.6%	0.569
No	0	
**History of surgery**		
Yes	27.3%	0.249
No	10.5%	
**Presence of comorbidity**		
Yes	14.3%	0.692
No	22.2%	
**Year of surgery**		
2016 or earlier	37.5%	0.040
After 2016	8.0%	
**Lymphotomy**		
Yes	22.2%	0.692
No	14.3%	
**Scope of surgery**		
Total PE	23.3%	0.412
Anterior PE and posterior PE	9.1%	
**Surgical duration, min**		
≤ 480	12.0%	0.225
> 480	31.3%	
**Maximum tumor diameter, cm**		
< 4	31.3%	0.235
≥ 4	13.0%	
**Pelvic wall resection**		
Yes	18.8%	1.000
No	20.0%	

### Adjuvant treatment

Twenty-one patients (51.2%) received adjuvant therapy after the operation, of which 14 (66.7%) received chemotherapy alone, 4 (19.0%) received concurrent radiotherapy and chemotherapy, and 3 (14.3%) received radiotherapy alone (Table 5).

**Table 5:** Detailed regimen of adjuvant treatment

Regimen of adjuvant treatment	n (%)
**Chemotherapy**	
Paclitaxel and cisplatin	10 (47.6)
Paclitaxel and cisplatin + PD-1 blockade	1 (4.8)
Lobaplatin	1 (4.8)
Nanoparticle albumin-bound paclitaxel + PD-1 blockade	1 (4.8)
Docetaxel + Lobaplatin	1 (4.8)
**Radiotherapy**	3 (14.3)
**Chemoradiotherapy**	4 (19.0)

### Survival outcomes

**Fig. 2:**
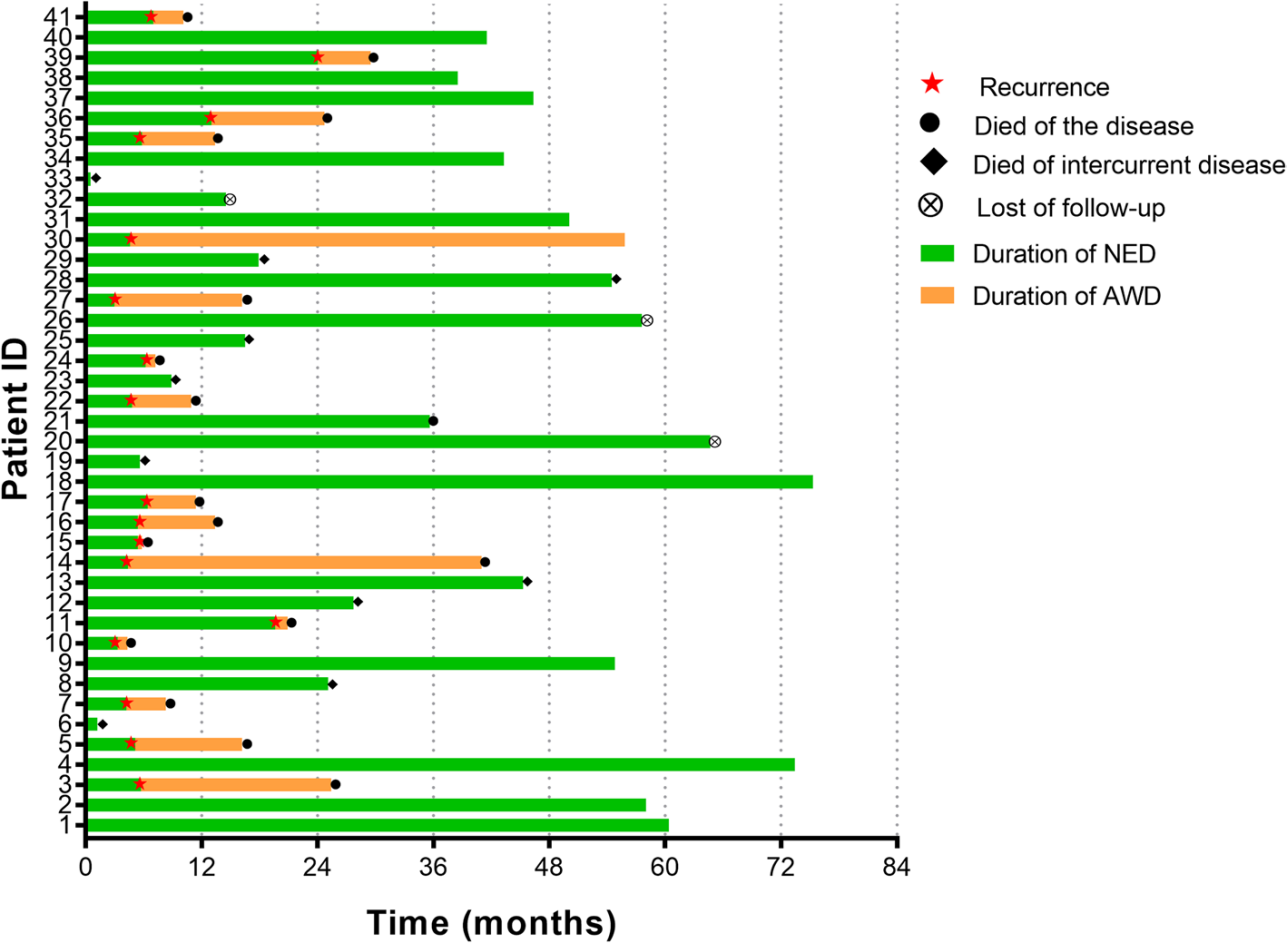
Swimmer plot of the patients received PE in this cohort (*n* = 41). Abbreviations: AWD, alive with disease; NED, no evidence of disease

The last follow-up date was May 10, 2023. The median follow-up time was 55.8 (range 1–75.3) months, and 3 cases (7.3%) were lost to follow-up. During the follow-up, 14 (31.4%) uncontrolled and 3 (7.3%) recurrent cases were observed. Extra-pelvic uncontrol/recurrence (8 cases, 47.1%) was the most common type and 2 intrapelvic uncontrol (11.8%) was observed. There were 7 patients (17.1%) had both extra and intra pelvic uncontrol/recurrence. Twenty-seven deaths (65.9%) were observed during the follow-up (Fig. 2).

**Fig. 3:**
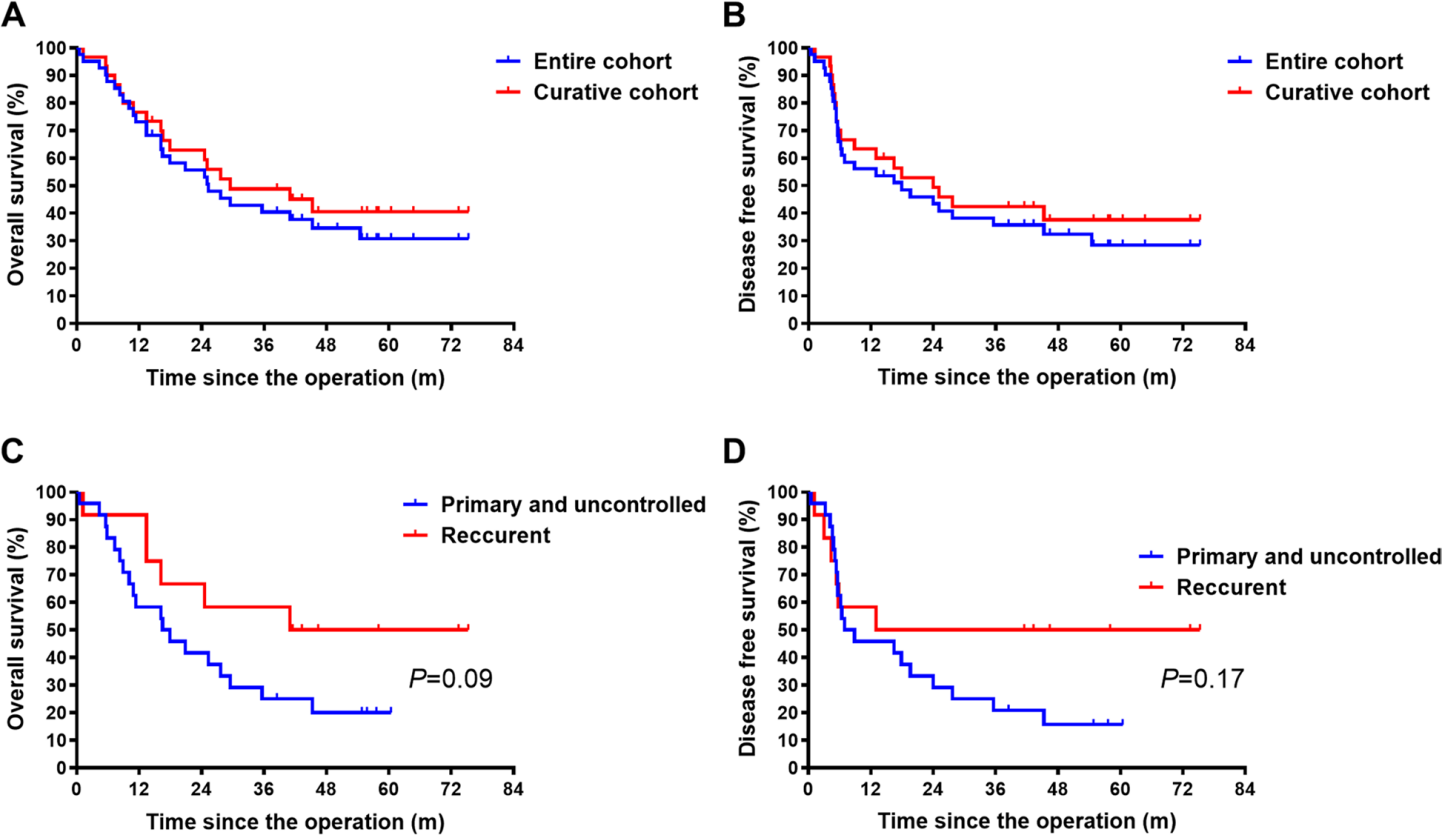
Survival curves of this cohort. Overall survival (**A**) and disease-free survival (**B**) for the entire cohort and patients received curative surgeries. Overall survival (**C**) and disease-free survival (**D**) for cervical cancer subgroups

For the entire cohort, the median DFS and OS were 17.9 months (95% CI 0–36.2 months) and 25.3 months (95% CI 14.8–35.8 months), respectively. And the 5-year DFS and OS were 28.5%, 30.8%. For patients underwent curative PE, (those with no macroscopic residual tumor and no pelvic wall involvement) the median DFS and OS were 24.0 months (95% CI 9.7–38.3 months) and 29.5 months (95% CI 5.5–53. 5 months). The 5-year DFS and OS was 37.6% and 40.6%, respectively (Fig. 3A and B).

Cervical cancer constituted the majority of our cohort, prompting us to conduct subgroup analyses on cervical cancer. Overall, 24 cases (58.5%) of primary cervical cancer were included, of which 22 were uncontrolled cases and there were also 12 (29.3%) cases of recurrent cervical cancer. Patients with recurrent cervical cancer had better OS and DFS compared to those with primary cervical cancer, although the difference was not statistically significant (Fig. 3C and D).

Among the patients who underwent LESS-APE, no uncontrol or recurrence was observed, but 1 patient died of other disease. As for the patients who underwent open surgery, there were one case (16.7%) had uncontrolled tumors and one case (16.7%) died of other disease.

## Discussion

Despite its high morbidity and mortality, pelvic exenteration remains a critical treatment option for patients with locally advanced gynecological tumors. Given the complexity and systemic nature of PE, involving multi-organ resection, the careful selection of candidates and the expertise of the surgical team are crucial in reducing the incidence of associated complications. Additionally, the advancement of surgical techniques contributes to improving the quality of the operation. Our current study suggested that PE was safe for patients evaluated through a multi-disciplinary treatment approach, with some achieving long-term survival post-procedure. We also observed that umbilical single-port laparoscopy may be a technically feasible option for APE, meriting further investigation.

The primary indication of PE is locally advanced or recurrent cervical cancer [14]. Cervical cancer was also the major disease in the current study, accounting for 87.8%. Consistent with this, cervical cancer constituted 87.8% of cases in our study, with most patients having a history of previous treatment, including 90.2% who received radiotherapy. This underscores that PE’s main role remains as palliative treatment for cervical cancer patients unresponsive to radiotherapy.

Previous studies [5, 15] have reported that about 7.7%-18% of patients found extra-pelvic metastases during the procedure and then finally gave up the operation. In the current study, only 2 cases (4.9%) of extra-pelvic metastases were found during the operation, suggesting that the preoperative screening and selection was strict. And multi-disciplinary team (MDT) assessment before the procedure is highly recommended.

Pelvic wall involvement was once considered a contraindication for PE due to the difficulty in achieving radical resection. However, assessing pelvic wall involvement based solely on gynecological examination can be subjective. Post-radiotherapy fibrosis of the pelvic wall tissues may affect preoperative evaluations. Several studies have indicated that patients with pelvic wall recurrence can still benefit from PE [8, 16, 17]. Jurado et al. [5] found that R0 resection was achieved in 28.6% of the patients with lateral recurrences and the 10-year disease-specific survival rate of those patients was 33.3%, which was not significantly different from that of patients with central recurrence. In a retrospective study of Höckel et al. [18], 67 cases in 91 patients with locally advanced or recurrent cervical cancer or vaginal cancer underwent laterally extended endopelvic resection (LEER) were found tumor fixed to the pelvic wall. And all the procedures were performed successfully, with a mortality and morbidity of 2% and 70% respectively, and the locoregional tumor control rate reached 92%. Their R0 resections involved extended pelvic wall muscle resection, aligning with the surgical principle in our study, which advocates for as extensive as possible removal of involved pelvic wall muscle to ensure negative margins, based on pelvic devascularization resection.

In our study, two patients with FIGO stage IV A cervical cancer underwent APE. These patients presented with vesicovaginal fistula and bilateral renal dysfunction, respectively. While surgery was not the first-line treatment option, after extensive discussions, both patients and their families opted for surgical intervention, seeking symptom relief. Postoperatively, they received adjuvant radiotherapy and chemoradiation, respectively. Up to the last follow-up, no recurrence was observed in these cases. The decision to perform PE in stage IV A cervical cancer patients remains a topic of debate [19–21]. A study by Marnitz et al. [20] showed that 43% of German surgeons would opt for PE in such cases. This percentage increases to 61% if pre-surgery tumor-related fistulas and severe local symptoms are present, with a 5-year overall survival rate of 52.5% when PE is utilized as the initial treatment modality [21]. Currently, platinum-based concurrent radiotherapy and chemotherapy remain the standard care for stage IV A cervical cancer. However, for certain patients suffering from fistulas and severe locoregional symptoms, PE, following thorough evaluation and detailed communication, stands as a viable and safe alternative.

Ovarian cancer, endometrial cancer and carcinosarcoma often have a tendency of distant metastasis. Whether PE should be performed for those patients remains controversial. In the current study, 1 patient underwent PPE due to recurrent endometrial adenocarcinoma, and 1 patient underwent APE due to recurrent endometrial stromal sarcoma. No preoperative distant metastasis or positive lymph node was found by physical examination and imaging examination, and both of them received radiotherapy after the surgery. No recurrence was found by the time of the last follow-up. Khoury-Collado et al. [22] suggested that PE was a feasible treatment for patients with endometrioid adenocarcinoma and low-grade sarcoma. Seagle et al. [23] showed that for patients of uterine malignancies undergoing PE, if the lymph nodes confirmed positive, the OS was significantly shorter than that of patients with negative lymph node. Similarly, the OS of patients with distant metastases was also significantly shorter than that of patients without distant metastases. In view of these evidences, positive lymph nodes and distant metastasis should be a contraindication for PE in such patients.

In the current study, the median operation time was similar to previous studies, while the median blood loss and length of postoperative hospital stay were comparatively lower [5, 20, 24, 25]. Notably, we found that the years of the operation was significantly related to the operation time, suggesting a learning curve for PE and emphasizing the importance of the surgical team’s experience. Previous studies have demonstrated that increased surgical team experience leads to reduced morbidity, shorter ICU stays, decreased costs, and lower perioperative mortality rates in medical centers with extensive PE experience [26, 27]. Such hospitals had more experience in complicated and major surgeries and most of them were teaching hospitals, which were armed with rich medical resources, so that they were more capable to deal with severe complications.

With the improvement of the medical care, the morbidity of PE and the perioperative mortality have gradually descended [3, 24, 28]. The morbidity of PE was 30% to 82% according to literature [7, 14, 16, 29, 30]. And the mortality has dropped to 2%-4% [31, 32]. In our study, the overall morbidity was 52.4%, with grade III and higher complications occurring in 24% of cases. The incidence of pelvic and abdominal infection was the highest in both short-term and long-term complications. One patient died 38 days after the procedure due to sepsis and MODS. Part of the patients (21.4%) had infections and fistulas before the surgeries, and the long operation time and large surgical wounds of PE may lead to the spread of infections. In addition, urinary tract or/and digestive tract reconstruction, drainage in the pelvic and abdominal for a long time, and the worsened nutritional status due to delayed post-surgery gastrointestinal recovery, are possibly related to the high incidence of infection after PE. Our findings indicate a significant association between the year of surgery and the occurrence of major complications, further highlighting the critical role of the surgical team's experience. With the improvement of surgical techniques, the increasingly strict selection of patients and more reasonable application of antibiotics, the incidence of perioperative infection has gradually decreased [33]. There was no perioperative death, which preliminarily showed that PE was safe and feasible for strictly selected patients.

Recently, laparoscopic PE has been performing with the intention to reduce the morbidity and laparoscopic and robotic surgeries have become one of the alternative methods for PE [9, 12, 34]. As Martínez et al. [11] reported, there was no significant difference between patients received laparoscopic and open PE in perioperative morbidity, operation time, the length of hospital stays, and OS, but patients received laparoscopic PE had less intraoperative blood loss and lower blood transfusion rate. The study of Bizzarri et al. [35] suggested that the perioperative morbidity of minimally invasive PE was lower than that of open PE and intraoperative blood loss was less. In addition, laparoendoscopic single site surgery has been widely used in gynecology. Besides the advantages of traditional laparoscopy, LESS has the advantages of better cosmetic effects, less puncture-related complications and postoperative pain. The incisions required for open PE are typically extensive, increasing the likelihood of wound complications. In contrast, trans-umbilical laparoendoscopic single-site (LESS) surgery involves a solitary incision at the umbilicus, potentially reducing the risk of such issues. In our current study, we successfully performed three trans-umbilical LESS anterior pelvic exenterations (APE) without any intraoperative complications. Notably, the intraoperative blood loss in these cases was significantly lower than in open APE. Furthermore, no recurrence was observed up to the last follow-up. To the best of our knowledge, this represented the first report of trans-umbilical LESS PE and further evidence is warranted to verify the feasibility. However, it becomes difficult to identify tumor and normal tissue owing to the lack of feedbacks in laparoscope, and thus sometimes it is hard to determine the range of resection. Therefore, the assessment of the relationship between the tumor and the pelvic wall is important. It is not suitable for tumors which is tightly fixed to the pelvic wall to receive laparoscopic PE. Patients with large residual lesions and severe pelvic-abdominal adhesions, as discovered during exploration, were not suitable candidates for LESS. For such patients, conversion to open surgery should be considered. Moreover, studies [36, 37] have pointed out that for patients with early-staged cervical cancer, the prognosis of receiving minimally invasive surgeries is worse than that of open surgeries. The effect of laparoscopic PE remains to be further observed and studied.

There were several limitations to our study. First, this study was a retrospective single-center study, which has inherent biases. Second, the sample size of the study was relatively small, limiting the generalizability of the findings. Lastly, this study included multiple cancer types, and further analysis specifically focusing on cervical cancer is needed.

## Conclusion

In this study, we conducted a retrospective analysis on the safety and long-term survival outcomes of pelvic exenteration (PE), as well as the feasibility of minimally invasive PE, in treating gynecologic malignancies at a single medical center in China. Our results suggested that PE was safe for patients assessed by the multi-disciplinary treatment. Some patients could achieve long-term survival after the procedure. In addition, our study provided preliminary evidence that umbilical single-port laparoscopy was a technically feasible approach for APE and further investigation was warranted.

### Supplementary Information

**Additional file 1: Table S1.** Details of prior treatments. **Table S2.** Detailed Clavien-Dindo Classification of Complications.

## Data Availability

The datasets used and analyzed in the current study are available from the corresponding author upon reasonable request. The authenticity of this article has been validated by uploading the key raw data onto the Research Data Deposit public platform (www.researchdata.org.cn).

## Abbreviations

PE: Pelvic exenteration
DFS: Disease free survival
OS: Overall survival
TPE: Total pelvic exenteration
APE: Anterior pelvic exenteration
PPE: Posterior pelvic exenteration
LESS: Laparoendoscopic single site
MDT: Multi-disciplinary team
LEER: Laterally extended endopelvic resection
