# Supplementary material for: Long-term outcomes of pelvic exenterations for gynecological malignancies: a single-center retrospective cohort study
Source: BMC Cancer. 2024 Jan 17;24:88. doi: 10.1186/s12885-024-11836-3 (PMC10792850; doi:10.1186/s12885-024-11836-3)
Supplement: Supplementary file 1 — Additional file 1: Table S1. Details of prior treatments. Table S2. Detailed Clavien-Dindo Classification of Complications. [file 12885_2024_11836_MOESM1_ESM.docx]

**Supplementary Tables**

**Table S1** Details of prior treatments

| **Prior Treatments** | **Amount（%）** |
| --- | --- |
| **Surgery** |  |
| Yes | 23（56.1%） |
| No | 18（43.9%） |
| **Radiotherapy** |  |
| Yes | 37（90.2%） |
| No | 4（9.8%） |
| **Regimen** |  |
| None | 2（4.9%） |
| Radiotherapy Only | 1（2.4%） |
| Surgery Only | 2（4.9%） |
| Radiotherapy +Chemotherapy | 16（39.0%） |
| Radiotherapy +Surgery | 2（4.9%） |
| Radiotherapy +Surgery +Chemotherapy | 18（43.9%） |

**Table S2** Detailed Clavien-Dindo Classification of Complications

| **Grade** | **Early complications（%）** | **Late complications（%）** |
| --- | --- | --- |
| I | 4（12.1%） | 3（17.6%） |
| II | 23（69.7%） | 8（47.1%） |
| III | 5（15.2%） | 6（35.3%） |
| IV | 0 | 0 |
| V | 1（3.0%） | 0 |
